# Supplementary material for: A Balance between Pro‐Inflammatory and Pro‐Reparative Macrophages is Observed in Regenerative D‐MAPS
Source: Adv Sci (Weinh). 2023 Feb 10;10(11):2204882. doi: 10.1002/advs.202204882 (PMC10104668; doi:10.1002/advs.202204882)
Supplement: Supplementary file 1 — Supporting Information [file ADVS-10-2204882-s001.pdf]

Supplementary Material for

A balance between pro-inflammatory and pro-reparative macrophages is observed in regenerative D-MAPS

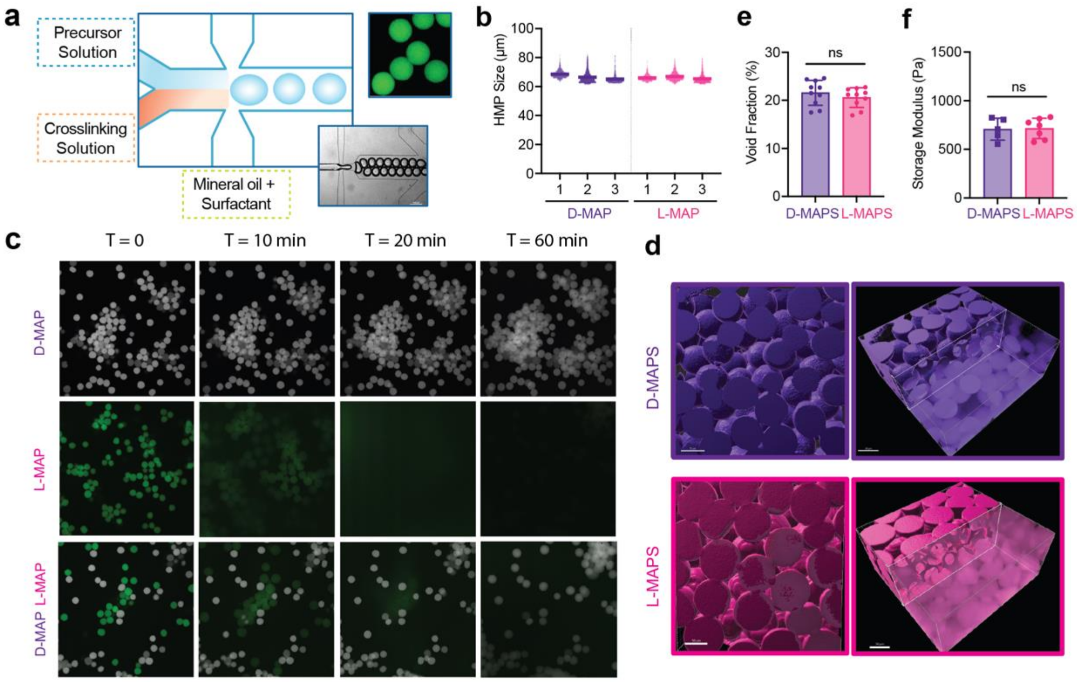

Supplementary Figure 1: L- and D-chiral peptide crosslinked microporous annealed particle scaffolds (MAPS) have similar mechanical properties. a, Scheme illustrating microgel formation using a microfluidic water-in-oil emulsion system. A precursor solution and a crosslinker solution were fused and segmented into droplets, which were then crosslinked into microgels via Michael addition. b, Microgels were monodisperse and around 70 µm in size. c, L-MAPS degraded within 20 minutes when incubated in collagenase IV solution at 37°C while D-MAPS took more than 1 hour to degrade. d, IMARIS software-rendered fluorescent images showing D-MAPS (top) and L-MAPS (bottom) with similar microstructures. e-f, both MAPS have comparable void fraction and storage modulus. Data are means ± SEM, n = 6, statistical significances were calculated using. Statistical analysis: Student's t-test between L- and D-MAPS groups. Error bars, mean ± s.e.m. n = 10 scaffolds per group in e, n = 5 for D-MAPS and n = 7 for L-MAPS in f.

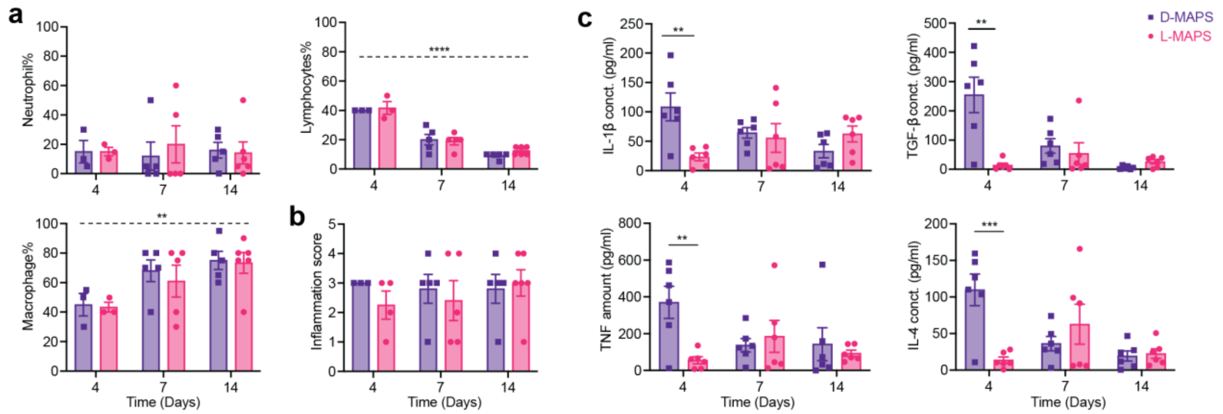

Supplementary Figure 2: D-MAPS elicited an early immune response on day 4. a, percentages of neutrophils, macrophages and lymphocytes among the total infiltrated cells inside the implants as a subjective quantification by a dermatopathologist based on H&E stained paraffin sections. The dotted line indicated a significant time-dependent difference in both scaffolds. b, histologic assessment of inflammation level in L- and D-MAPS based on a modified 5-point scoring system established and agreed upon by two dermatopathologists. c, ELISA results of cytokine level inside the hydrogel implants from a repeated subcutaneous implantation study. Statistical analysis: two-way ANOVA with Šídák's multiple comparisons test made between L- and D-MAPS groups only when there was a significance in the interaction term of scaffold type x time. * $p<0.05$, ** $p<0.01$, *** $p<0.001$, **** $p<0.0001$. Error bars, mean ± s.e.m. n = 6 mice per group for all assays with some data points removed due to experimental reasons.

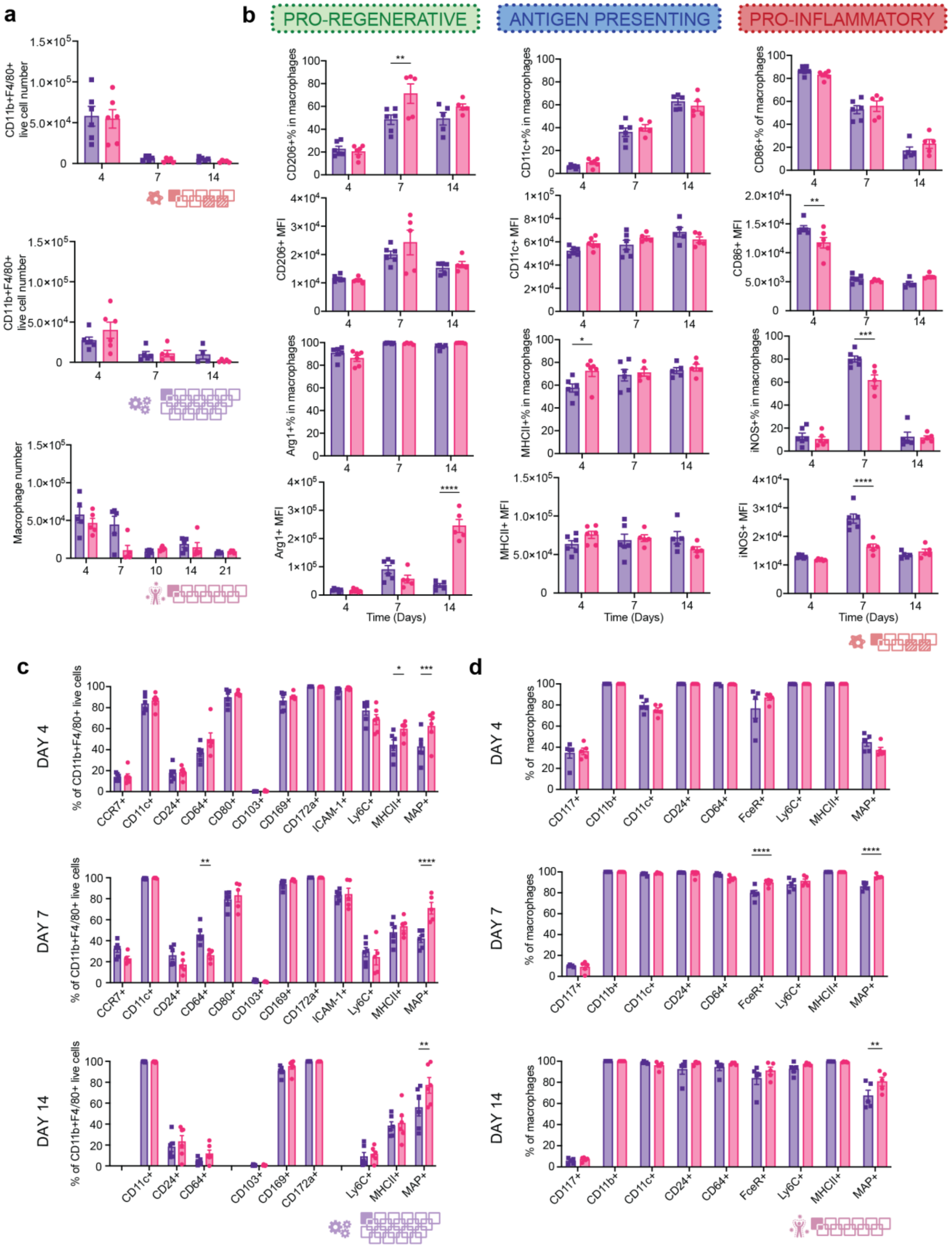

Supplementary Figure 3: Macrophage phenotypes characterized by different gating strategies (traditional gating versus algorithm-aided gating) and by different flow cytometry panels. a, CD11b+F4/80+ cell number or pre-gated macrophage number over time. The red, purple, pink

symbols beneath the graphs stand for the 9-color macrophage panel, the 20-color APC panel, the 11-color innate panel used to acquire the data. b, traditional gating of the data set presented in Figure 3, showing the positive expression percentage and MFI of Arg1, CD206, CD11c, MHCII, CD86 and iNOS in CD11b+F4/80+ macrophage population over time. c, the marker expression profile in CD11b+F4/80+ macrophage population over time. d, the marker expression profile in pre-gated macrophage population (following the corresponding gating strategy) over time. Statistical analysis: two-way ANOVA with Šídák's multiple comparisons test made between L- and D-MAPS groups only when there was a significance in the interaction term of scaffold type x time. * p<0.05, ** p<0.01, *** p<0.001, **** p<0.0001. Error bars, mean ± s.e.m. n = 6 mice per group for all assays with some data points removed due to experimental reasons.

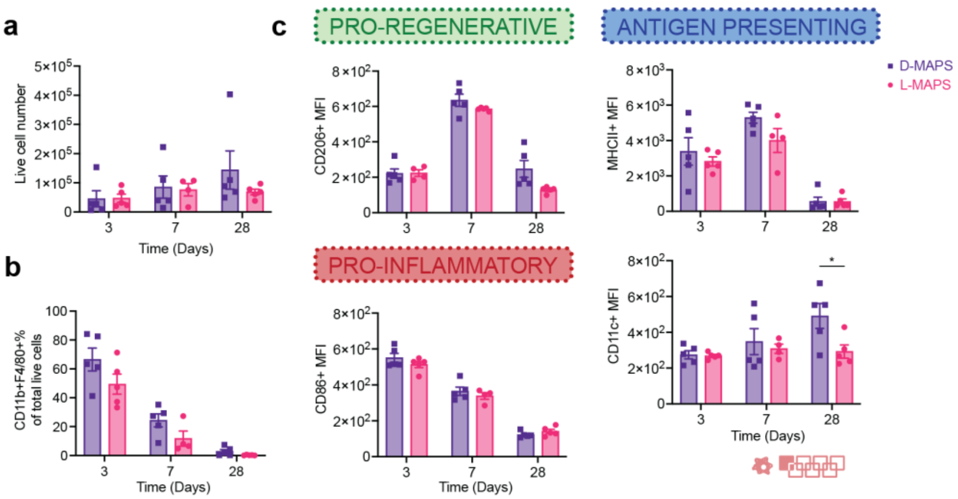

Supplementary Figure 4: Macrophage phenotypes characterized by a 7-color panel showed a similar trend of phenotype shift over time. a, total live cell number over time. b, percentage of CD11b+F4/80+ cells in total live cells over time. c, traditional gating of the data set, showing the MFI of CD206, CD11c, MHCII, CD86 in CD11b+F4/80+ macrophage population over time. Statistical analysis: two-way ANOVA with Šídák's multiple comparisons test made between L- and D-MAPS groups only when there was a significance in the interaction term of scaffold type x time. * p<0.05. Error bars, mean ± s.e.m. n = 5 mice per group. The red symbol beneath the graph stands for the 7-color macrophage panel used to acquire the data.

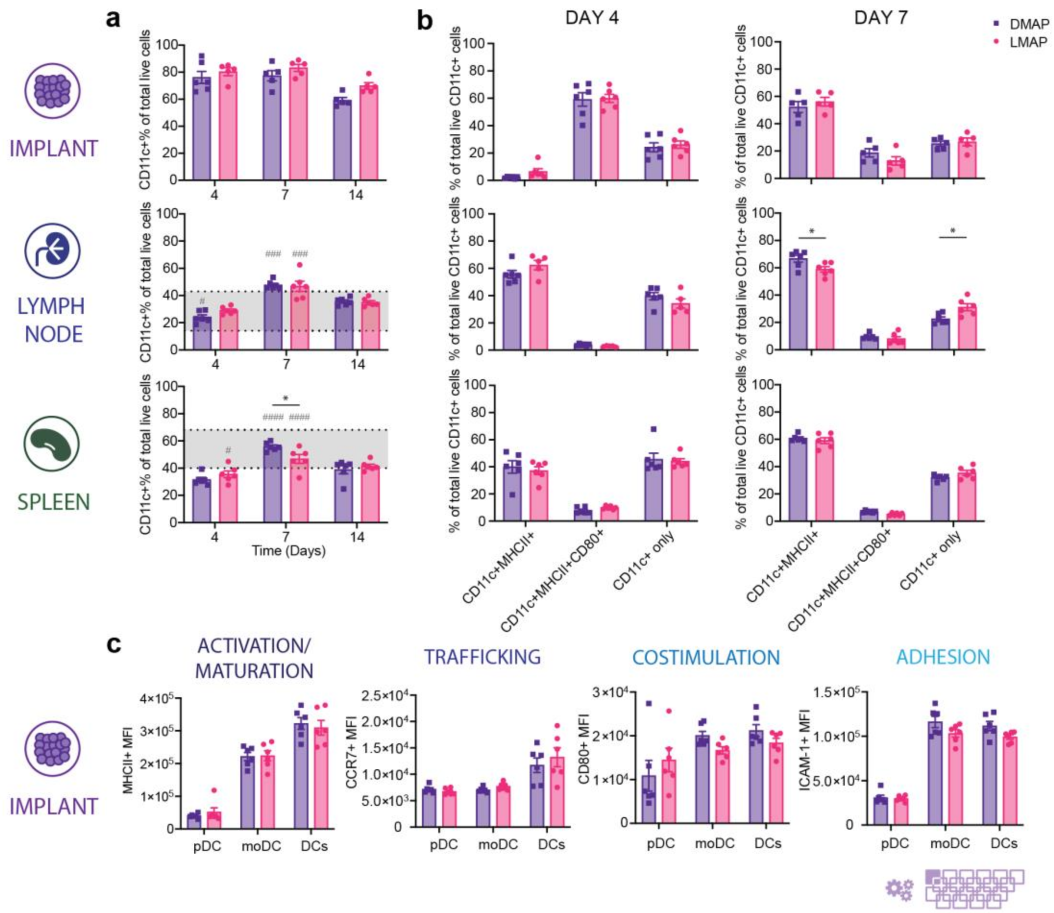

Supplementary Figure 5: MAP+CD11c+ cells migrated from the implant sites to draining lymph nodes and the spleen and showed mature antigen-presenting phenotype. a, the percentage of CD11c+ cells among the total live cells infiltrating the implants, residing in dLN or spleen. The gray shade stands for the baseline level of CD11c+% in dLN and the spleen in health naïve mice. b, the percentages of CD11c+MHCII+, CD11c+MHCII+CD80+ (activated mature APCs) and CD11c+ cells among CD11c+ live cells on day 4 and day 7. c, MFI of MHCII, CCR7, CD80 and ICAM-1 in pDC, moDC and cDC on day 4. Statistical analysis: two-way ANOVA with Šídák's multiple comparisons test made between L- and D-MAPS groups only when there was a significance in the interaction term of scaffold type x time. After a two-way ANOVA, Dunnet method was used to compare the experiment groups with the baseline control group. * $p<0.05$, **/## $p<0.01$, ***/### <0.001, ****/#### <0.0001. Asterisks stand for comparisons between L- and D-MAPS. Pound signs stand for comparisons between L- or D-MAPS and the baseline control (mice without implant). Error bars, mean ± s.e.m. n = 6 mice per group. The purple symbol in the bottom left corner of the graph stands for the 20-color APC panel used in this figure.

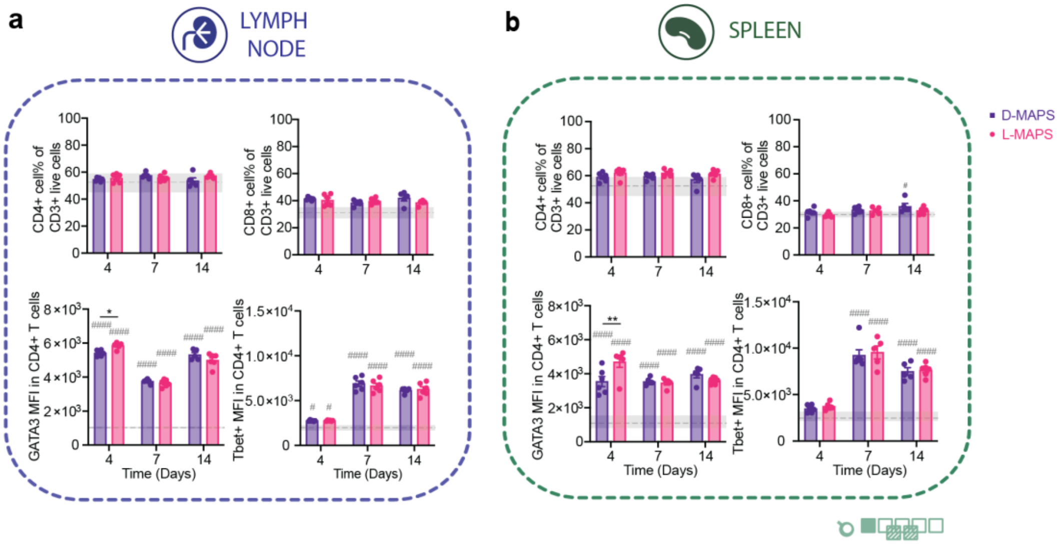

Supplementary Figure 6: MAPS induced a phenotype switch in T cell population compared to baseline in draining lymph nodes and spleen of mice with D or L-MAPS across 3 time points. Each column shows the percentage of specific cell subpopulations. Data are means ± SEM, n = 6, and statistical significances were calculated using two-way ANOVA with the Sidak method to compare D vs. L-MAPS (black asterisk) and the Dunnet method to compare each treatment against the baseline control group without material implantation (gray asterisk). */# p<0.05, ** p<0.01, #### p<0.0001. The green symbol in the bottom right corner stands for the 7-color T cell panel used in this figure.

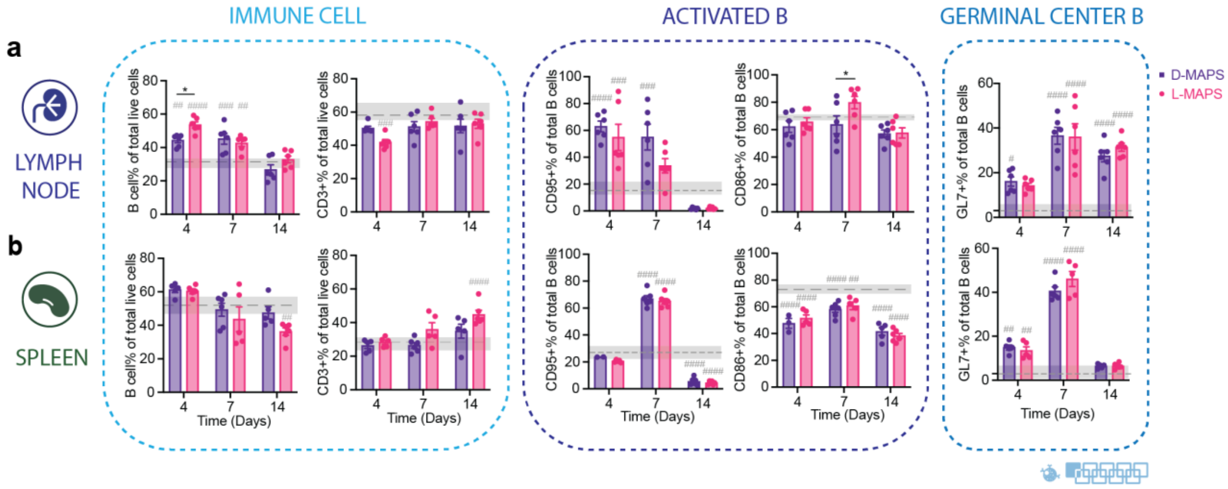

Supplementary Figure 7: MAPS induced a phenotype switch in B cell population compared to baseline in draining lymph nodes and spleen of mice with D or L-MAPS implants across 3 time points. Each column shows the percentage of specific cell subpopulations. Data are means ± SEM, n = 6, and statistical significances were calculated using two-way ANOVA with the Sidak method to compare D vs. L-MAPS (black asterisk) and the Dunnet method to compare each treatment against the baseline control group without material implantation (gray number sign). */# p<0.05, **/## p<0.01, ***/### p< 0.01, ****/#### p<0.0001. The blue symbol in the bottom right corner stands for the 9-color B cell panel used in this figure.

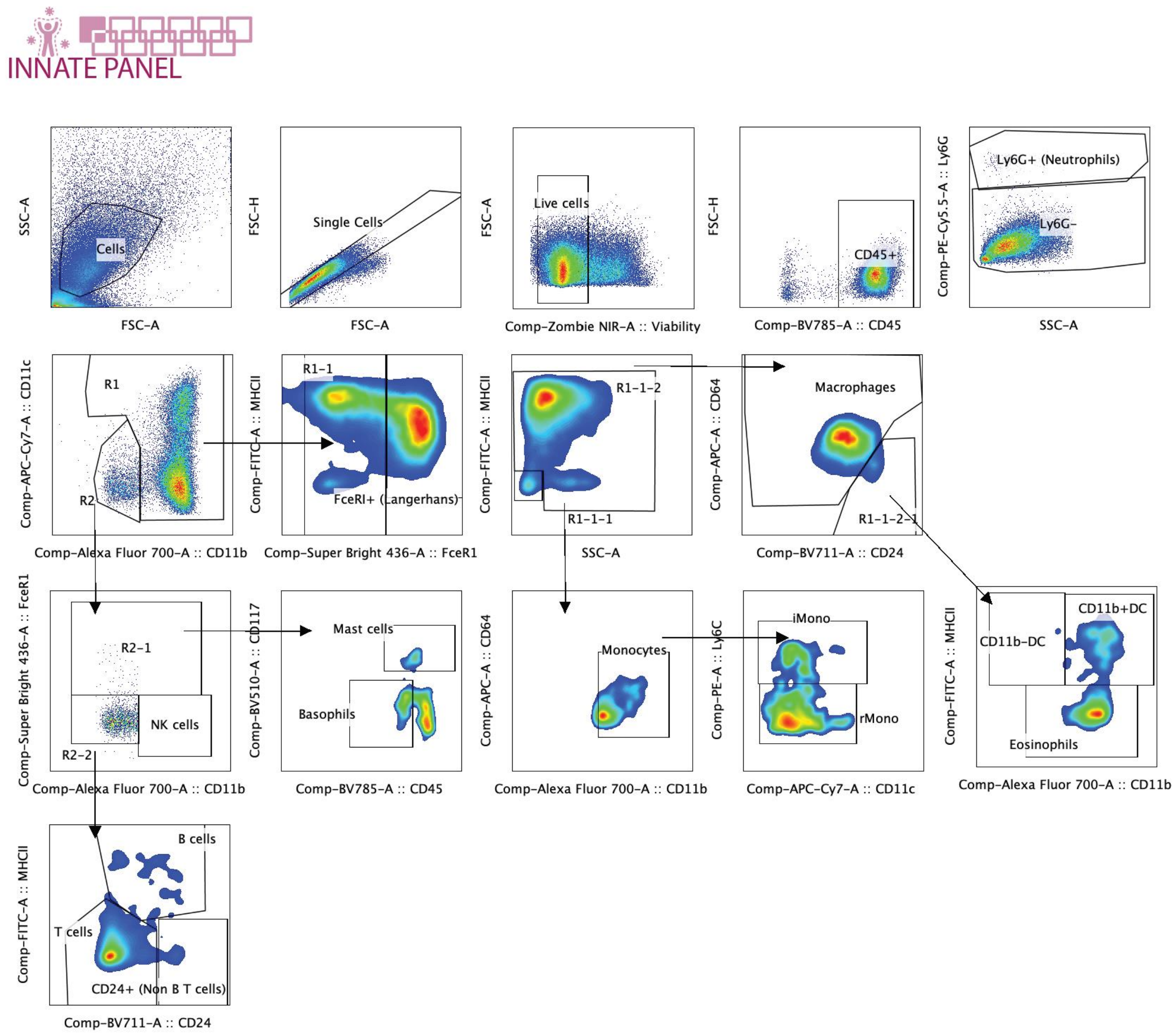

Supplementary Figure 8: Gating strategy for flow cytometry analysis on myeloid cells with 11 markers, using one representative MAPS sample as an example. Adapted panel from Yu, et al.[1]

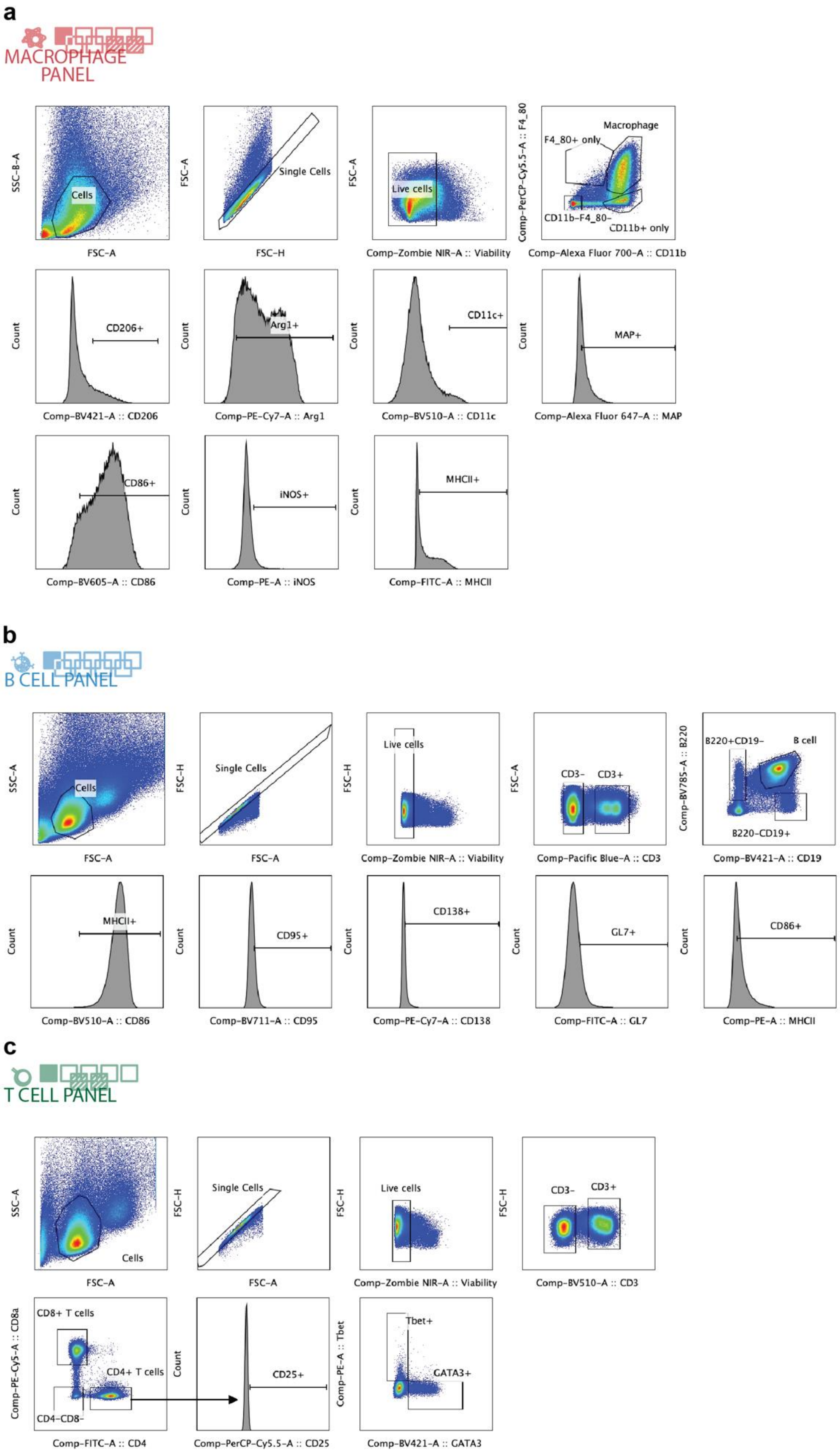
a
MACROPHAGE PANEL
SSC-B-A
FSC-A
Cells
FSC-A
FSC-H
Single Cells
FSC-A
Comp-Zombie NIR-A :: Viability
Live cells
Comp-PerCP-Cy5.5-A :: F4_80
Comp-Alexa Fluor 700-A :: CD11b
Macrophage
F4_80+ only
CD11b-F4_80-
CD11b+ only
Count
CD206+
Comp-BV421-A :: CD206
Arg1+
Comp-PE-Cy7-A :: Arg1
CD11c+
Comp-BV510-A :: CD11c
MAP+
Comp-Alexa Fluor 647-A :: MAP
CD86+
Comp-BV605-A :: CD86
iNOS+
Comp-PE-A :: iNOS
MHCII+
Comp-FITC-A :: MHCII
b
B CELL PANEL
SSC-A
FSC-A
Cells
FSC-H
Single Cells
Comp-Zombie NIR-A :: Viability
Live cells
Comp-Pacific Blue-A :: CD3
CD3-
CD3+
Comp-BV785-A :: B220
Comp-BV421-A :: CD19
B220+CD19-
B cell
B220-CD19+
Count
MHCII+
Comp-BV510-A :: CD86
CD95+
Comp-BV711-A :: CD95
CD138+
Comp-PE-Cy7-A :: CD138
GL7+
Comp-FITC-A :: GL7
CD86+
Comp-PE-A :: MHCII
c
T CELL PANEL
SSC-A
FSC-A
Cells
FSC-H
Single Cells
Comp-Zombie NIR-A :: Viability
Live cells
Comp-BV510-A :: CD3
CD3-
CD3+
Comp-PE-Cy5-A :: CD8a
Comp-FITC-A :: CD4
CD8+ T cells
CD4+ T cells
CD4-CD8-
Count
CD25+
Comp-PerCP-Cy5.5-A :: CD25
Comp-PE-A :: Tbet
Comp-BV421-A :: GATA3
Tbet+
GATA3+

Supplementary Figure 9: Gating strategy for flow cytometry analysis on: a) macrophages with 9 markers, using one representative MAPS sample as an example. b) B cells with 9 markers, using one representative splenocyte sample as an example. c) T cells with 7 markers, using one representative splenocyte sample as an example.

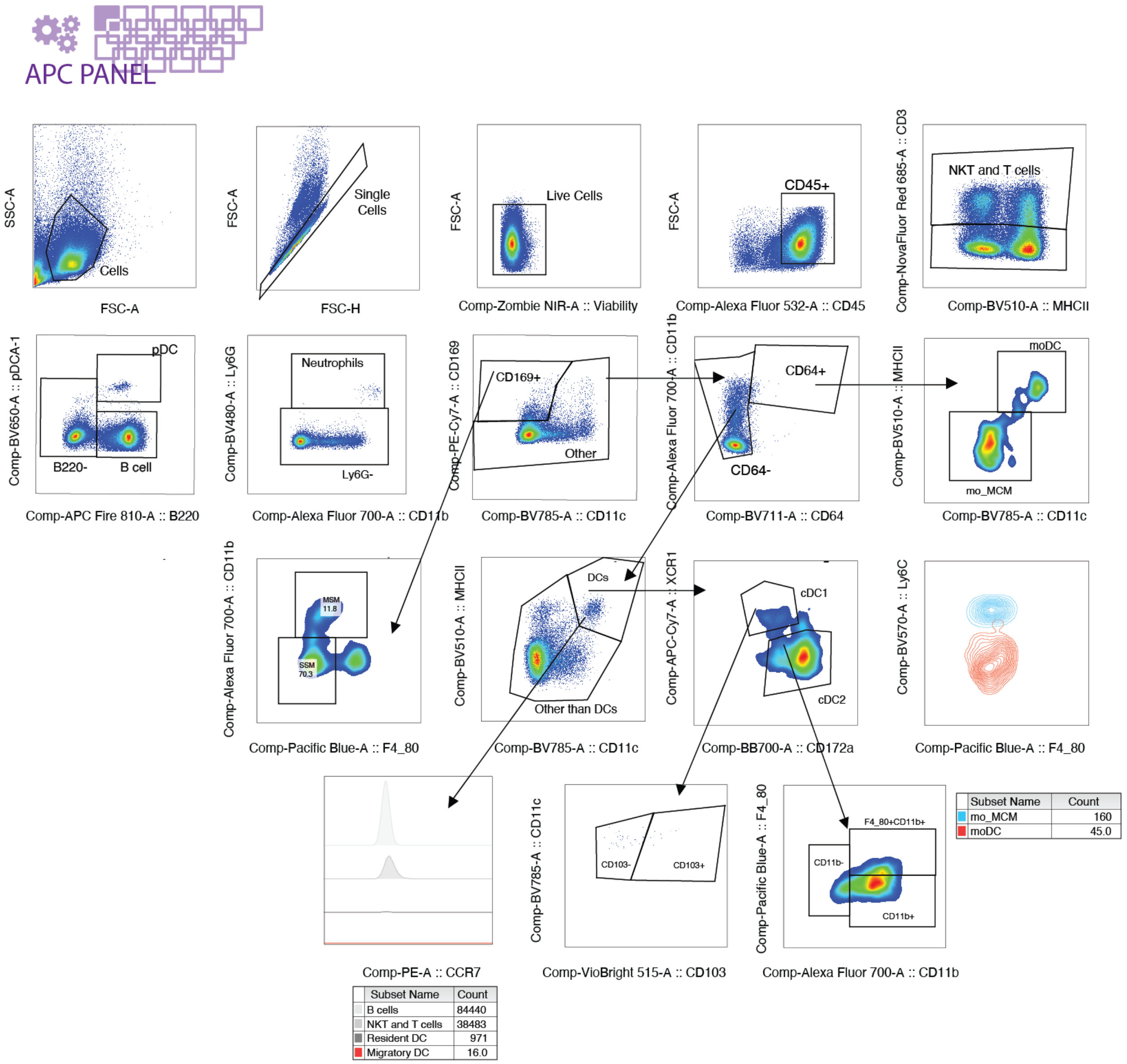

Supplementary Figure 10: Gating strategy for flow cytometry analysis on APCs with 20 markers, using one representative spleen as an example. OMIP-061 adapted from DiPiazza, et al. [2]

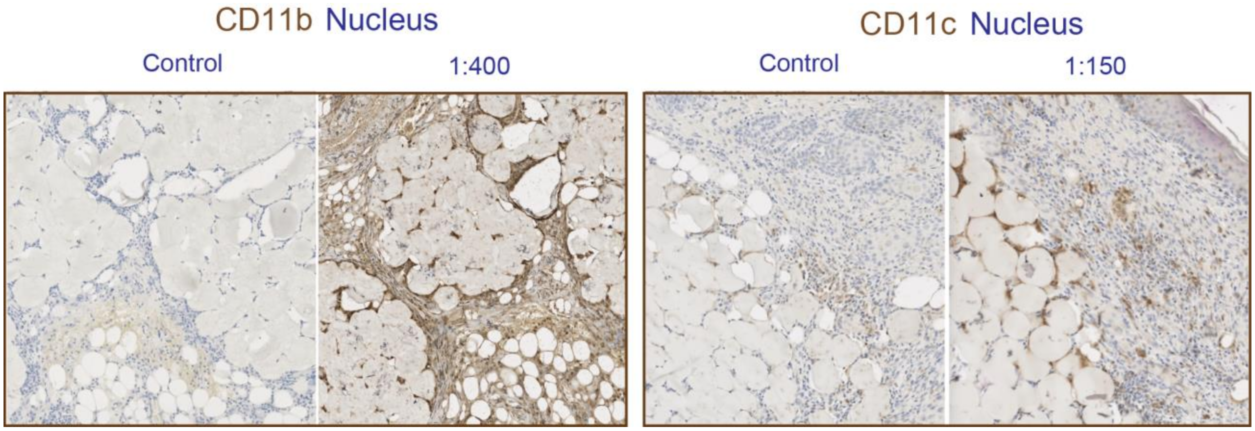

Supplementary Figure 11: primary delete control for CD11b and CD11c IHC stains.

Supplementary Table 1. *Scoring of Granulation Tissue/Vascularization*

Score	Criteria
0	No granulation tissue.
1	Early granulation tissue, no vascularization.
2	Mature granulation tissue, early vascularization.
3	Mature granulation tissue with mature blood vessel formation.

Supplementary Table 2. *Scoring of Collagen Deposition/Fibroplasia*

Score	Criteria
0	No collagen deposition/fibroplasia.
1	Fibroblast proliferation/no collagen deposition.
2	Fibroblast proliferation with minimal collagen deposition.
3	Fibroblast proliferation with extensive haphazard collagen deposition.
4	Extensive organized collagen deposition or complete replacement of dermis with fibrous tissue (mature scar).

Supplementary Table 3. *Scoring of Inflammation*

Score	Criteria
0	No inflammatory cells.
1	1-50 leukocytes per high power field.
2	51-100 leukocytes per high power field.
3	101-250 leukocytes per high power field.
4	>250 leukocytes per high power field or micro-abscesses or abscesses present.

Supplementary Table 4. A list of antibodies for the innate panel

Marker	Clone	Fluorophore	Supplier	Cat. #	Titration (tissue/cell type)
FCeR1	MAR-1	PB	BioLegend	134314	2 in 100 (Splenocyte)
CD117	ACK2	PE	BioLegend	135105	1.25 in 100 (Splenocyte)
CD45	30-F11	BV785	BioLegend	103149	0.625 in 100 (Implant)
CD24	M1/69	BV711	BD Biosciences	563450	0.25 in 100 (Implant)
Ly6C	HK1.4	BV510	BioLegend	128033	1.25 in 100 (Splenocyte)
MHCII	M5/114.15.2	SB600	eBioscience	63-5321-82	0.5 in 100 (Splenocyte)
Ly6G	1A8	PE-Cy5	eBioscience	15-9668-82	0.3125 in 100 (Implant)
CD64	X54-5/7.1	PE-Cy7	BioLegend	139313	0.625 in 100 (Implant)
CD11b	M1/70	Alexa Fluor 700	eBioscience	56-0112-80	0.0625 in 100 (Implant)
CD11c	N418	APC-Cy7	BioLegend	117323	0.039 in 100 (Implant)
		Zombie NIR	BioLegend	423105	0.0625 in 100 (Splenocyte)

Supplementary Table 5. A list of antibodies for the macrophage panel

Marker	Clone	Fluorophore	Supplier	Cat. #	Titration (cell type)
CD206	C068C2	BV421	BioLegend	141717	2 in 100 (Macrophage)
CD11c	N418	BV510	BioLegend	117338	1.25 in 100 (Macrophage)
MHCII	M5/114.15.2	FITC	eBioscience	11-5321-82	0.25 in 100 (Macrophage)
iNOS	CXNFT	PE	eBioscience	12-5920-80	1.5 in 100 (Macrophage)
F4/80	BM8	PerCP-Cy5.5	eBioscience	45-4801-80	0.5 in 100 (Macrophage)
CD86	GL-1	BV605	BioLegend	105037	0.156 in 100 (Macrophage)
Arg1	A1exF5	PE-CY7	eBioscience	17-3697-80	1.5 in 100 (Macrophage)
CD11b	M1/70	Alexa Fluor 700	eBioscience	56-0112-80	0.25 in 100 (Macrophage)
Viability		Zombie NIR	BioLegend	423105	0.0625 in 100 (Macrophage)

Supplementary Table 6. A list of antibodies for the B cell panel

Marker	Clone	Fluorophore	Supplier	Cat. #	Titration (cell type)
CD3	17A2	PB	BioLegend	100214	0.25 in 100 (Splenocyte)
GL7	GL7 (RUO)	FITC	BD Pharmingen	553666	1 in 100 (Splenocyte)
CD95	Jo2 (RUO)	BV711	BD Biosciences	740716	1 in 100 (Splenocyte)
B220	RA3-6B2	BV785	BioLegend	103226	0.0625 in 100 (Splenocyte)
CD19	1D3	BV421	BioLegend	115537	0.3125 in 100 (Splenocyte)

MHCII	M5/114.15.2	BV510	BioLegend	107608	0.125 in 100 (Splenocyte)
CD138	281-2	PE-Cy7	BioLegend	142513	1 in 100 (Splenocyte)
CD86	GL-1	PE	BioLegend	105040	0.625 in 100 (Macrophage)
Viability		Zombie NIR	BioLegend	423105	0.0625 in 100 (Splenocyte)

Supplementary Table 7. A list of antibodies for the T cell panel

Marker	Clone	Fluorophore	Supplier	Cat. #	Titration (cell type)
CD3	17A2	BV510	BioLegend	100233	1.25 in 100 (Splenocyte)
CD4	GK1.5	FITC	BD Pharmingen	557307	0.156 in 100 (Splenocyte)
CD8a	53-6-7	PE-Cy5.5	BioLegend	100710	0.1 in 100 (Splenocyte)
CD25	PC61	PerCP-Cy5.5	BioLegend	102030	0.5 in 100 (Splenocyte)
Tbet	4B10	PE	BioLegend	644810	2.5 in 100 (Splenocyte)
GATA3	16E10A23	BV421	BioLegend	653806	2.5 in 100 (Splenocyte)
Viability		Zombie NIR	BioLegend	423105	0.0625 in 100 (Splenocyte)

Supplementary Table 8. A list of antibodies for the APC panel

Marker	Clone	Fluorophore	Supplier	Cat. #	Titration (cell type)
CD3	17A2	NovaFluor Red 685	Thermo Fisher	M002T02R02	0.0625 in 100 (Splenocyte)
CD45	30-F11	Alexa Fluor 532	Thermo Fisher	58-0451-82	0.3125 in 100 (Splenocyte)
XCR1	ZET	APC-Cy7	BioLegend	148223	0.3125 in 100 (Splenocyte)
CD64	X54-5/7.1	BV711	BioLegend	139311	0.156 in 100 (Splenocyte)
Ly6C	HK1.4	BV570	BioLegend	128030	0.625 in 100 (Splenocyte)
CD169	3D6.112	PE-Cy7	BioLegend	142412	2.5 in 100 (Splenocyte)
F4/80	BM8	PB	Thermo Fisher	MF48028	2 in 100 (Splenocyte)
CD80	16-10A1	PE-CF594	BioLegend	104737	1.25 in 100 (Splenocyte)
CD103	M290	VioBright515	Miltenyi	130-111-609	2 in 100 (Splenocyte)
CD24	M1/69	SB600	Thermo Fisher	63-0242-80	0.625 in 100 (Splenocyte)
CD11c	N418	SB780	Thermo Fisher	78-0114-82	0.625 in 100 (Splenocyte)
pDCA-1	927	BV650	BioLegend	127019	0.078 in 100 (Splenocyte)
ICAM-1	3E2	SB436	Thermo Fisher	62-0542-80	0.625 in 100 (Splenocyte)
CCR7	4B12	PE	Thermo Fisher	12-1971-80	1.25 in 100 (Splenocyte)
B220	RA3-6B2	APC/Fire810	BioLegend	103277	0.3125 in 100 (Splenocyte)
CD172a	P84	BB700	BD Bioscience	742205	1 in 100 (Splenocyte)
MHCII	M5/114	BV510	BioLegend	107635	0.25 in 100 (Splenocyte)
Ly6G	1A8	BV480	BD Bioscience	746448	0.125 in 100 (Splenocyte)
CD11b	M1/70	Alexa Fluor 700	eBioscience	56-0112-80	0.0625 in 100 (Splenocyte)
Viability		Zombie NIR	BioLegend	423105	0.03125 in 100 (Splenocyte)

Supplementary Table 9 fluorescence minus one (FMO) controls macrophage panel

Phenotype gating	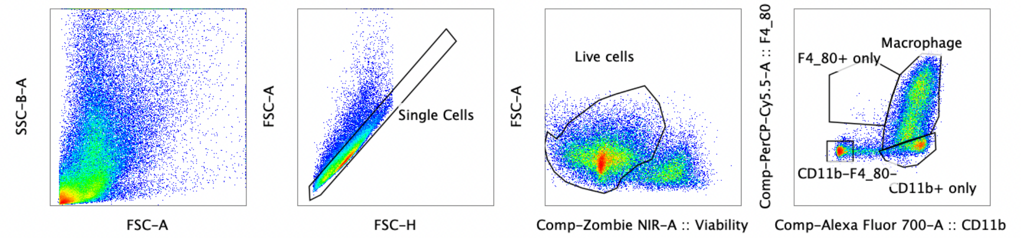
FMO-MHCII (Gated on macrophage population)	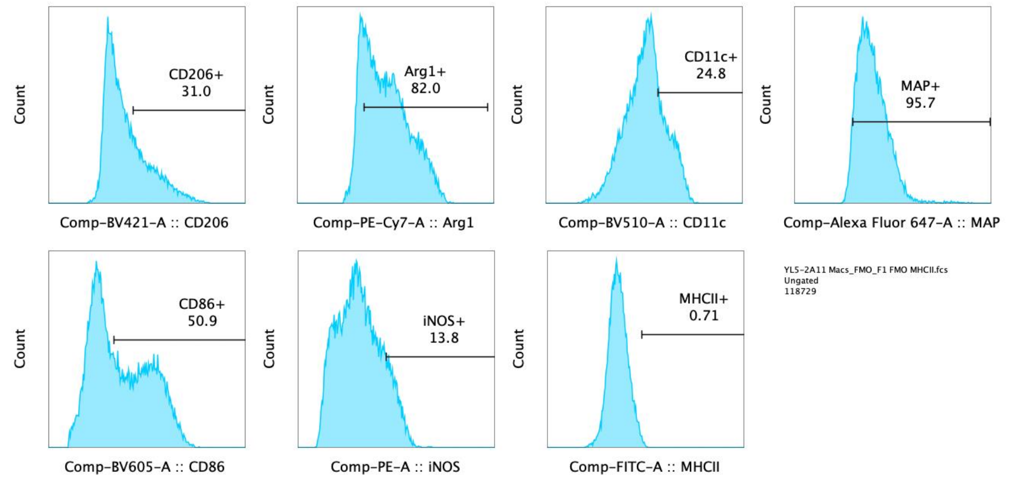
FMO-CD86 (Gated on macrophage population)	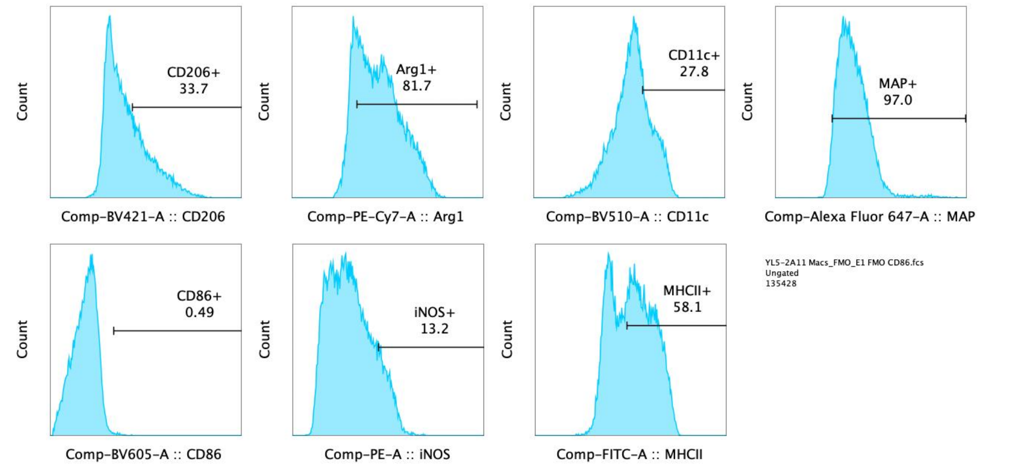

FMO-iNOS (Gated on macrophage population)

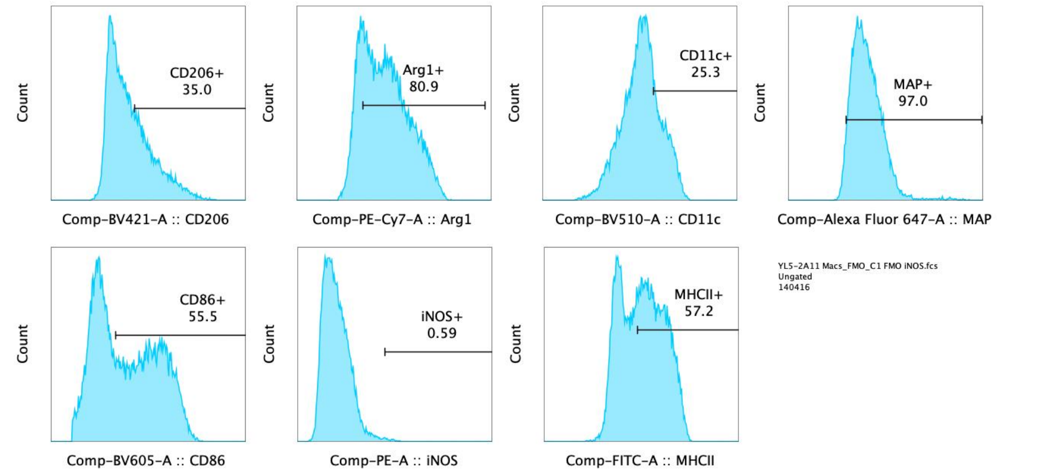

FMO-CD11c (Gated on macrophage population)

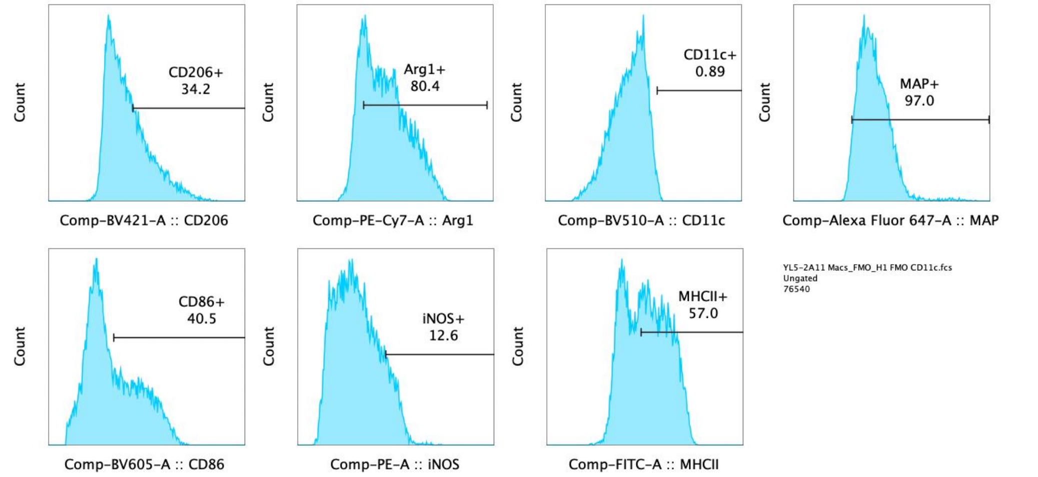

FMO-CD206 (Gated on macrophage population)

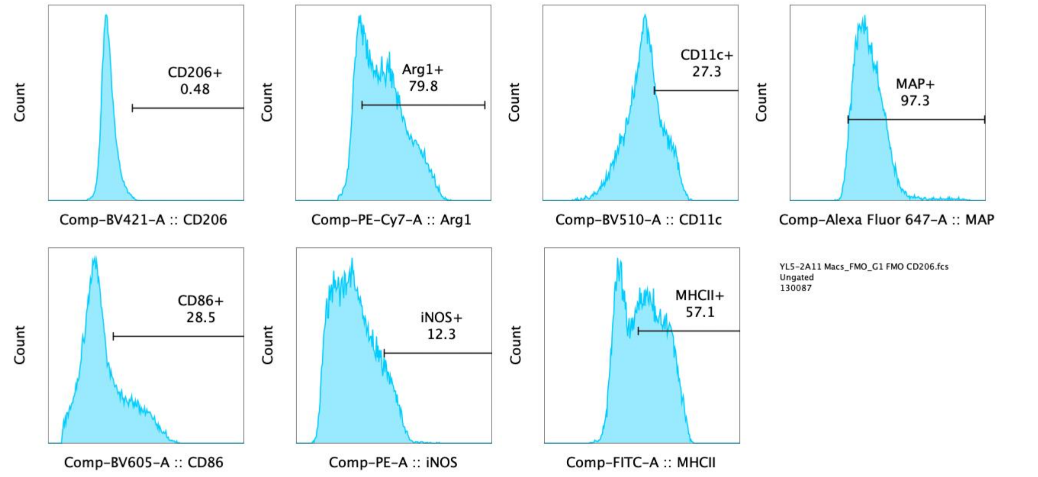

<table>
<tr>
<td>FMO-Arg1 (Gated on macrophage population)</td>
<td>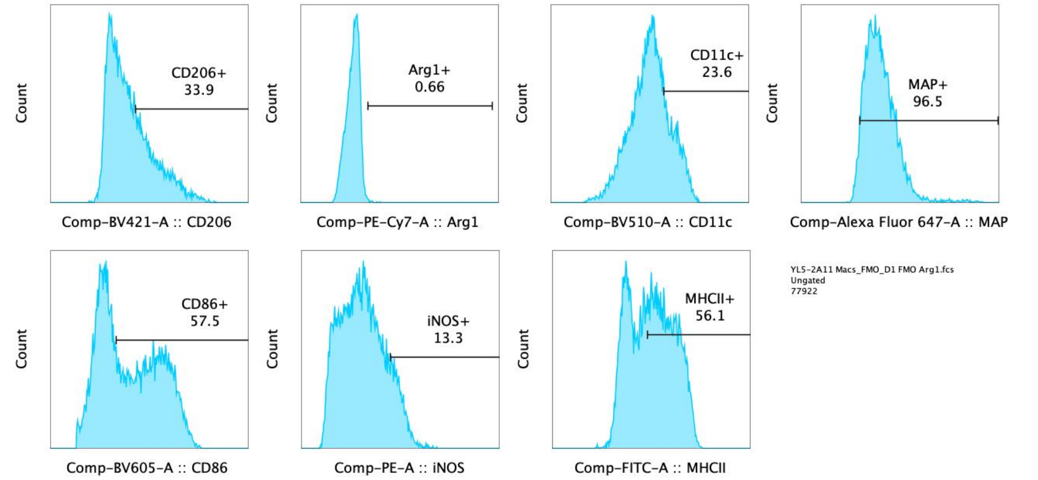
</td>
</tr>
<tr>
<td>FMO-MAP AF647 (Gated on macrophage population, using L-MAPS implant without AF647 labeling)</td>
<td>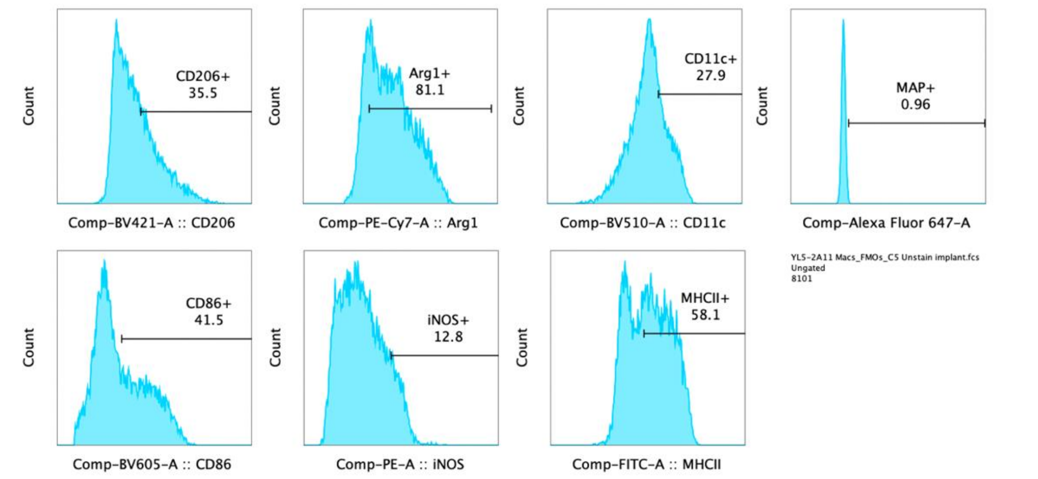
</td>
</tr>
</table>

Supplementary Table 10 fluorescence minus one (FMO) controls B cell panel

<table>
<tr>
<td>Phenotype gating</td>
<td>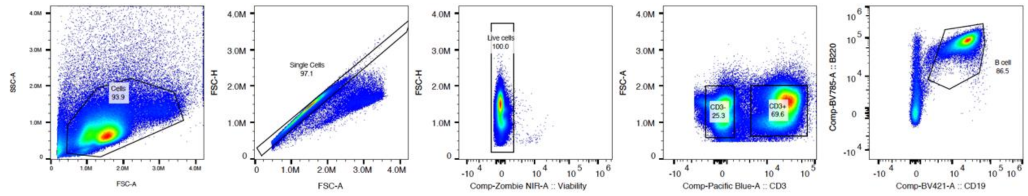
</td>
</tr>
<tr>
<td>FMO-MHCII (Gated on B cell population)</td>
<td>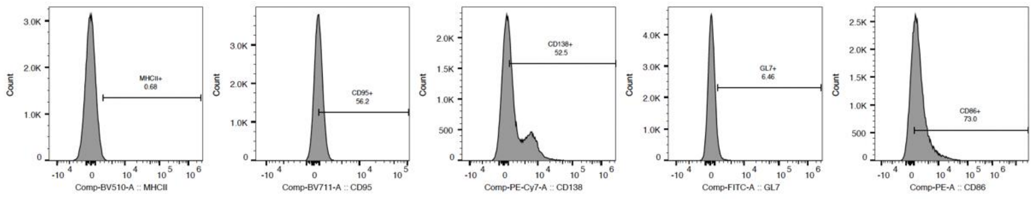
</td>
</tr>
</table>

FMO-Tbet

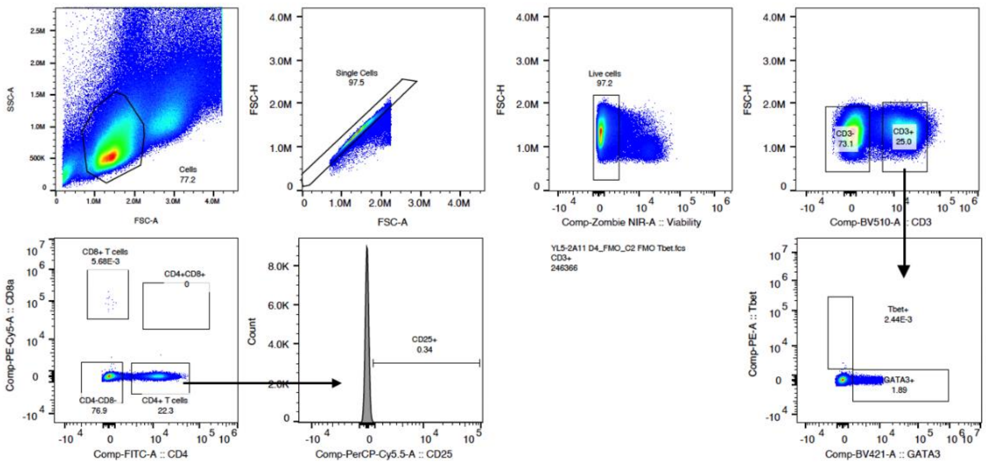

FMO-CD95 (Gated on B cell population)

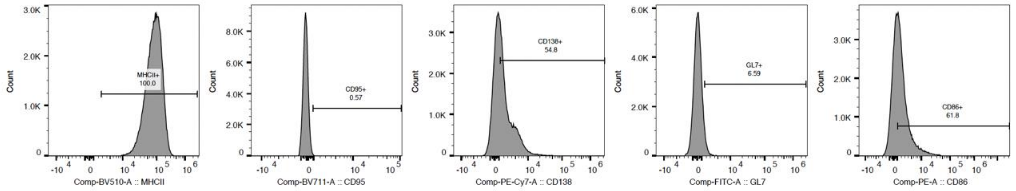

FMO-CD138 (Gated on B cell population)

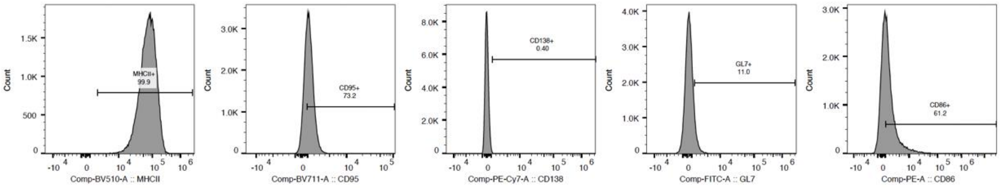

FMO-GL7 (Gated on B cell population)

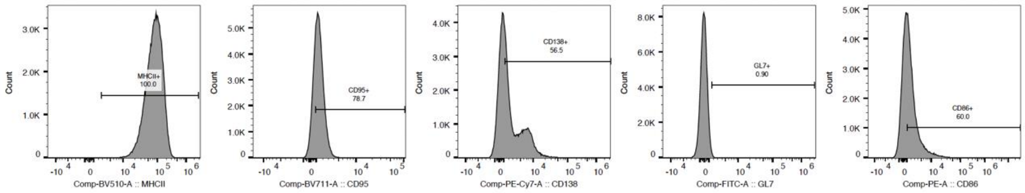

FMO-CD86 (Gated on B cell population)

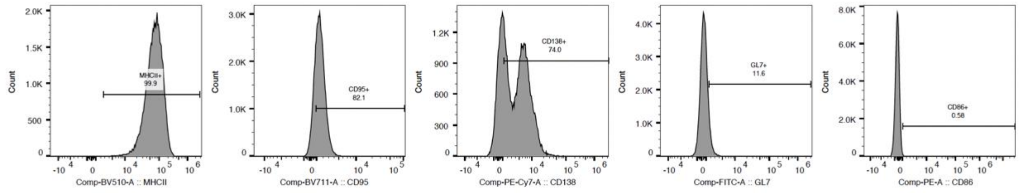

<table>
<tr><td>FMO-
GATA3</td><td>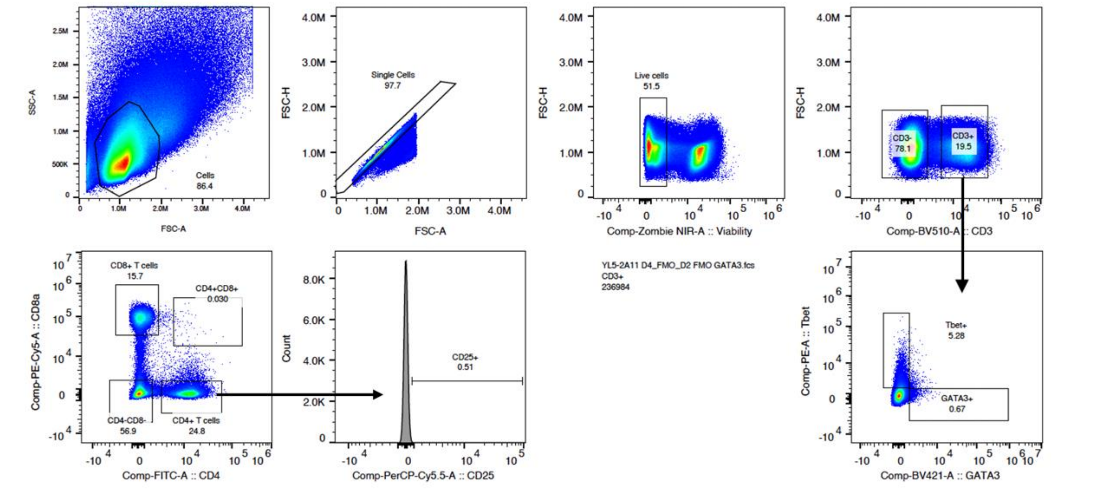
</td></tr>
<tr><td>FMO-
CD25</td><td>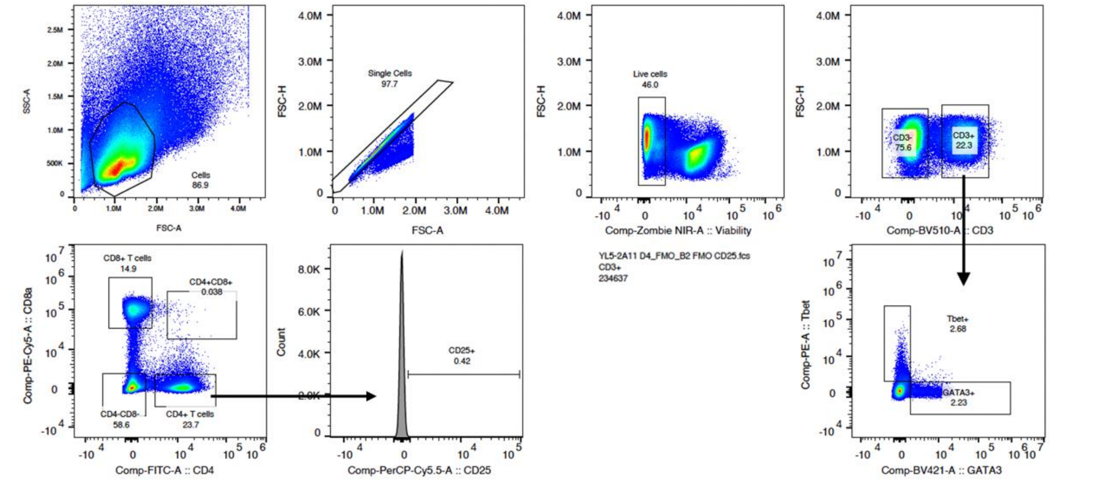
</td></tr>
</table>

Supplementary Table 11. fluorescence minus one (FMO) controls T cell panel

References

1 Yu, Y.-R. A. *et al.* A protocol for the comprehensive flow cytometric analysis of immune cells in normal and inflamed murine non-lymphoid tissues. *PloS one* **11**, e0150606 (2016).

2 DiPiazza, A. T., Hill, J. P., Graham, B. S. & Ruckwardt, T. J. OMIP-061: 20-color flow cytometry panel for high-dimensional characterization of murine antigen-presenting cells. *Cytometry Part A* **95**, 1226-1230 (2019).
